# Evaluation of Zebrafish DNA Integrity after Individual and Combined Exposure to TiO_2_ Nanoparticles and Lincomycin

**DOI:** 10.3390/toxics10030132

**Published:** 2022-03-08

**Authors:** Filomena Mottola, Concetta Iovine, Marianna Santonastaso, Vincenzo Carfora, Severina Pacifico, Lucia Rocco

**Affiliations:** 1 Department of Environmental, Biological and Pharmaceutical Sciences and Technologies, University of Campania “Luigi Vanvitelli”, 81100 Caserta, Italy; concetta.iovine@unicampania.it (C.I.); vinci.carfora91@gmail.com (V.C.); severina.pacifico@unicampania.it (S.P.); lucia.rocco@unicampania.it (L.R.); 2 Department of Woman, Child and General and Special Surgery, University of Campania “Luigi Vanvitelli”, 80138 Napoli, Italy; marianna.santonastaso@unicampania.it

**Keywords:** environmental drug contamination, titanium dioxide nanoparticles, lincomycin, genotoxicity, DNA fragmentation

## Abstract

Environmental contamination by nanoparticles (NPs) and drugs represents one of the most debated issues of the last years. The aquatic biome and, indirectly, human health are strongly influenced by the negative effects induced by the widespread presence of pharmaceutical products in wastewater, mainly due to the massive use of antibiotics and inefficient treatment of the waters. The present study aimed to evaluate the harmful consequences due to exposure to antibiotics and NPs, alone and in combination, in the aquatic environment. By exploiting some of their peculiar characteristics, such as small size and ability to bind different types of substances, NPs can carry drugs into the body, showing potential genotoxic effects. The research was conducted on zebrafish (*Danio rerio*) exposed in vivo to lincomycin (100 mg/L) and titanium dioxide nanoparticles (TiO_2_ NPs) (10 µg/L) for 7 and 14 exposure days. The effects on zebrafish were evaluated in terms of cell viability, DNA fragmentation, and genomic template stability (GTS%) investigated using Trypan blue staining, TUNEL assay, and the random amplification of polymorphic DNA PCR (RAPD PCR) technique, respectively. Our results show that after TiO_2_ NPs exposure, as well as after TiO_2_ NPs and lincomycin co-exposure, the percentage of damaged DNA significantly increased and cell viability decreased. On the contrary, exposure to lincomycin alone caused only a GTS% reduction after 14 exposure days. Therefore, the results allow us to assert that genotoxic effect in target cells could be through a synergistic effect, also potentially mediated by the establishment of intermolecular interactions between lincomycin and TiO_2_ NPs.

## 1. Introduction

Over the last 20 years, pharmaceuticals have been the most investigated pollutants [1]. 

Lincomycin is an antibiotic belonging to the class of lincosamides, and it has been frequently used in human and veterinary medicine as a bacteriostatic and protein synthesis inhibitor in anaerobic bacteria [2]. It is a drug easily soluble in water and is chemically stable both in the dry state and in solution. 

Lincomycin is partially metabolized in the liver and both the drug and metabolites are excreted in limited quantities in urine and in larger quantities in bile and feces. Its metabolism is not well-defined, though the primary product recovered in humans after administration is unchanged lincomycin [3]. 

As it is widely used in the treatment of respiratory tract infections and in the treatment of toxoplasmosis and pneumocystis in patients with acquired immune deficiency syndrome (AIDS) [4], concerns, for both aquatic organisms and humans, have been raised about its disposal and its persistence in the environment. 

Although the amount used was relatively low (8 tons/year) in Korea as of 2003, this drug has been detected frequently in Korean surface waters at medium concentrations (maximum concentration) of 0.03–0.17 μg L^−1^ (2.66 μg L^−1^) in the major rivers [5]. Lincomycin has also been frequently detected around the world, e.g., 0.012 μg L^−1^ (0.355 μg L^−1^) in Southern Ontario, Canada [6]; 0.06 μg L^−1^ (0.73 μg L^−1^) in US waters [7]; 0.073 μg L^−1^ (0.249 μg L^−1^) in the Po and the Lambro, Italy [8]; and 23.81 ng/L in the Weihe River, China [9]. 

The continuous and rapid development of the pharmaceutical industry and the increase in the consumption of antibiotics require the utmost attention from the relevant authorities as their impact on the environment contributes to soil and water contamination and their impact on humans compromises the state of health. 

When discharged into sewage, drugs can continue to act in different forms on new substrates. Recent studies have shown that antibiotics, in compositions and concentrations like those found in the environment [10], can have some ecotoxic effects [11,12]. 

One of the major concerns is a possible interaction of drugs with other contaminants capable of aggregating with them and forming chemically more active compounds. In this scenario, nanomaterials (NMs), known to be widespread in the environment due to their extensive use in several industrial sectors, could interact with drugs and cause a synergistic effect on exposed organisms [13,14]. 

Among the NMs, titanium dioxide nanoparticles (TiO₂ NPs), widely used in the cosmetic, pharmaceutical, and food industries [15], were considered physiologically inert and to have few risks for humans, but further studies confirmed the toxicity of the aforementioned NPs in living organisms [16,17]. Furthermore, it has been shown that TiO₂ NPs plates of different sizes, regardless of the route of administration, can be translocated to the nervous system, accumulating and promoting morphological alterations and oxidative stress in neural cells through reactive oxygen species (ROS) production [18,19]. TiO_2_ NPs can induce cytotoxic, genotoxic, and carcinogenic responses both in vitro and in vivo [20,21]. In an aqueous culture medium, TiO_2_ NPs exhibited a stable dispersion with a remarkably low rate of aggregation sedimentation, causing genomic instability, loss of DNA integrity, and a high apoptosis rate in *Danio rerio* [22]. However, there is evidence that high TiO_2_ NPs concentrations may not generate effects or mild effects. In fact, 1 mg/L TiO_2_ NPs has no effect on hatching, survival, malformation rate of zebrafish larvae, and embryonic development [23].

A recent study on general and organ TiO_2_ NPs toxicity showed that they have no adverse effects up to a dose of 1000 mg/kg of body weight per day, probably due to the low absorption of these NPs; however, they can accumulate in the body due to their long half-life and induce DNA strand breaks and chromosomal damage, but not genetic mutations [24]. In fish, TiO₂ NPs can have systemic effects following the absorption of the gills and its distribution to the muscles, and although the evidence is not abundant, some studies have shown that TiO₂ NPs, given their small size, can directly penetrate cells and further act as a vehicle for other substances, including antibiotics, inducing damage at the level of genetic material [25,26,27]. However, based on literature studies, it was not possible to identify a cutoff value for the size of TiO_2_ NPs in relation to genotoxicity, as well as there was no evident correlation between the physicochemical properties of the TiO_2_ NPs and the potential genotoxicity as contradictory results [23]. At the same time, it is not easy to establish the environmental impact of this nanomaterial as predicted concentrations of TiO_2_ NPs in the environment are challenging to detect, in fact, the expected low concentrations range from 2 to 700 ng/L [28]. 

The ability of TiO₂ NPs to bind with and transport substances to cells has been exploited in nanomedicine to deliver drugs to target organs [29,30]; indeed, many drugs, such as lincomycin, have titanium dioxide added as a coloring to the capsule. 

Lincomycin showed genotoxicity on zebrafish erythrocytes and hepatocytes and induced an increase in DNA migration, as highlighted by the comet assay, and an increase in the micronucleus frequency [31]. 

On the other hand, our recent data showed that in vitro exposure to lincomycin did not produce DNA damage in human amniotic cells, leaving us to assume a different molecular behavior in vivo and in vitro, depending on the endpoint investigated; meanwhile, its co-exposure with TiO_2_ NPs caused a significant increase in DNA fragmentation and apoptosis, leading to the hypothesis that TiO_2_ NPs can influence the activity of lincomycin by increasing its bioavailability [32]. 

In the present study, for the first time, we assessed the cytotoxic and genotoxic effects caused by TiO_2_ NPs and lincomycin co-exposure on zebrafish (*Danio rerio*) in vivo. Zebrafish is a model organism which shares about 75% of its genome with humans. It has already been used in many genetic toxicology studies as a bioindicator [33] and as a predictive tool for analysis of biodistribution, controlled release and therapeutic results of nanopharmaceuticals and to facilitate the study of interactions between NPs, drugs, and biological systems [34].

The study was performed to highlight the effect of the two substances alone and in combination in terms of alterations in cell viability evaluated using Trypan Blue staining, in genomic stability evaluated using the random amplification of polymorphic DNA (RAPD) PCR technique, and in DNA integrity evaluated using the TUNEL assay.

## 2. Materials and Methods

### 2.1. Chemicals

TiO_2_ NPs (Aeroxide) were supplied by Evonik Degussa (Essen, Germany; lot No. 614061098). Aeroxide is certified 99.9% pure and is a blend of 75% rutile and 25% anatase forms with a dimensional average of 21 nm and a spheroid irregular shape [35]. The preparation of the TiO_2_ NP stock solution (10.0 mg/L) was performed according to the literature [32]. Briefly, the TiO_2_ NPs solution was ultrasonicated to disperse NPs and eliminate agglomeration. Sonication was carried out in ultrapure water (Millipore) for 3 h (40 kHz frequency, Dr. Hielscher UP 200S, Germany). Lincomycin (CAS 7179-49-9, 99% purity) was provided by Sigma-Aldrich (St. Louis, MO, USA). This product was provided as delivered and specified by the issuing pharmacopoeia. All the substances tested were dissolved in 10% DMSO and 90% H_2_O Milli-Q (dimethyl sulfoxide, CAS No. 67-68-5; Sigma-Aldrich, St. Louis, MO, USA) to give a final DMSO concentration of not more than 0.5%. Lincomycin is soluble in both water and DMSO. We chose DMSO as a solvent to mimic the same conditions as TiO_2_ NPs. 

### 2.2. Experimental Design 

Experiments were performed on 80 adult individual zebrafish, obtained from a local source (CARMAR sas, San Giorgio a Cremano, Italy). The specimens, without distinction of sex, were divided between four tanks equipped with a pump system to ensure the movement of water avoiding sedimentation as much as possible, containing 5 L of water, without a filter system, in which the selected substances were dissolved. The concentrations of TiO_2_ NPs (10 μg/L) were established based on previous studies which showed a higher genotoxic effect at the highest concentrations tested [22]. According to the literature data, no effect for aquatic organisms was induced by TiO_2_ at concentrations around 1 μg/L [36]. 

To have effective data on the drug toxicity, in order to generate concerns about its use and diffusion, the effect of high concentrations of lincomycin (100 mg/L) compared to those found in the environment (846 ng/L) was studied on the basis of the previous study comparing the effect of the high dose with that found in the environment which was able to induce genotoxic damage only at very prolonged exposure times [22,31]. 

The treatment was carried out for two different exposure times, 7 and 14 days, during which the effects of the test substances were monitored. The experimental design was as follows: negative control (NC): 20 zebrafish specimens (10 per tank) were bred in water supplemented with 0.5% DMSO; TiO_2_ NPs: 20 zebrafish specimens (10 per tank) were exposed to 10 μg/L TiO_2_ NPs; lincomycin: 20 zebrafish specimens (10 per tank) were exposed to 100 mg/L lincomycin; lincomycin + TiO_2_ NPs: 20 zebrafish specimens (10 per tank) were co-exposed to 10 μg/L TiO_2_ NPs and 100 mg/L lincomycin. The water with the dissolved substances was renewed every 7 days. The pH of the water was measured for all experimental conditions using pH meter PL700 (Eurotek, Milano, Italy).

At the end of each treatment time (7 and 14 days), blood cells were collected for each experiment. The experiments complied with the ARRIVE guidelines and were carried out in accordance with the UK Animals (Scientific Procedures) Act 1986 and the associated guidelines, EU Directive 2010/63/EU for animal experiments [37]. In detail, the zebrafish were anaesthetized with tricaine methyl sulfonate (Sigma-Aldrich, St. Louis, MO, USA) according to the Guide for Use and Care of Laboratory Animals (European Communities Council Directive), and efforts were made to minimize animal suffering and reduce the number of specimens used. 

At the end of the treatments described, about 25 μL of blood were taken from each fish by sampling below the gills using heparinized syringes. The blood cells were then mixed with phosphate-buffered saline (1× PBS, Thermo Fisher Scientific, Waltham, MO, USA) and subsequently centrifuged at 2000 revolutions per minute (rpm) for 10 min [31].

### 2.3. Lincomycin Quantitation in Tank Water

In order to investigate the fate of lincomycin and TiO_2_ NPs, UV–vis spectra were acquired using a Cary 100 UV–Vis spectrophotometer (Agilent Technologies Italia S.P.A., Cernusco s/N, Milan, Italy) in the range of 190–500 nm. Chromatographic analysis was carried out using an HPLC 1260 INFINITY II system (Agilent, Santa Clara, CA, USA) equipped with an Agilent G7129A autosampler, an Agilent GY115A DAD UV–visible detector, and an Agilent G711A quaternary pump. Separation was achieved using a Phenomenex Luna Phenyl-Hexyl 150 mm × 2 mm ID column (3.0 μm particle size) using a gradient of water (A) and acetonitrile (B), both with 0.1% formic acid. Starting with 10% B, a linear gradient was followed by 25% B for 6.0 min and then held at 25% B for a further 1.0 min. Finally, the starting conditions were restored and the system was re-equilibrated for a further 1 min. The total analysis time was 8.0 min, and the flow rate was 0.3 mL min^−1^. The injection volume was 5.0 μL.

### 2.4. Trypan Blue Staining

Cell viability was assessed using Trypan Blue (Thermo Fisher Scientific, Waltham, MA, USA) staining. A cell suspension was mixed with 0.4% dye. Finally, the mixture of blood and dye was placed on a slide and observed under an optical microscope (Optika XDS-3LT trinocular inverse microscope, Ponteranica, Italy) in order to carry out counting of dead cells compared to the vital ones. Trypan Blue selectively colored dead cells. 

### 2.5. TUNEL Assay

DNA fragmentation was determined using an In Situ Cell Death Detection Kit (Roche Diagnostics, Basel, Switzerland). Blood cell suspension (15.0 μL) previously washed in 1× PBS were placed on glass slides, fixed in 4% paraformaldehyde (Sigma-Aldrich, St. Louis, MO, USA) for 1 h at room temperature (RT), and air-dried. After 2 min of incubation in a permeabilizing solution, the TUNEL reaction mixture was placed on slides. Each slide was incubated for 1 h at 37 °C, stained with 4′,6-diamidino-2-phenylindole (DAPI, Sigma-Aldrich, St. Louis, MO, USA) solution for 5 min, and analyzed under a fluorescence microscope (Nikon Eclipse E-600, Minato, Tokyo, Japan) equipped with 330–380 nm BP and 420 nm LP filters. We analyzed 300 cells per slide, distinguishing those with fragmented DNA (green fluorescence) from those with intact DNA (blue fluorescence). The DNA fragmentation index (DFI) was calculated as the percentage of green nuclei out of all nuclei.

### 2.6. Genomic DNA Isolation and the RAPD PCR Technique

DNA isolation from zebrafish blood cells was performed using a commercial kit (High Pure PCR Template Preparation Kit, ROCHE Diagnostics, Basel, Switzerland) according to the manufacturer’s suggestions. The DNA purity and its concentration were evaluated using a Nanodrop 2000 (Thermo Fisher Scientific, Waltham, MA, USA). 

The RAPD PCR technique is simple, sensitive, and effective in identifying DNA damage by means of random amplification of fragments using PCR [38]. Briefly, PuREtaq Ready-To-Go PCR (Sigma-Aldrich, St. Louis, MO, USA), which contains nucleotides (dNTPs) and Taq DNA recombinant polymerase (2.5 units), was used. The DNA (40 ng) and primer 6 (5-d[CCCGTCAGCA]-30) (5 pmol µL^−1^) (Invitrogen, Waltham, MA, USA) were added in a final reaction volume of 25 µL. Primer 6 was selected for its previously tested high efficiency in amplifying the zebrafish DNA template [38]. 

The amplification reaction followed this cyclic program: one initial step (5 min at 95 °C), then 45 cycles comprising 1 min at 95 °C, 1 min at 36 °C, and 2 min at 72 °C. The reaction products were analyzed by means of electrophoresis on 2% agarose gel and examined after gel staining with 10× ethidium bromide (Sigma-Aldrich, St. Louis, MO, USA). The polymorphic pattern generated by the RAPD PCR profiles allowed the calculation of the genomic template stability (GTS%) as follows:

GTS = (1 − a/n) × 100

where a is the number of polymorphic bands detected in exposed groups and n is the total number of bands in the untreated group. Polymorphisms in the RAPD profiles included the loss of bands and gain of new bands with respect to the control. The GTS% was calculated for each experimental group exposed to different treatments, and changes in genomic stability are expressed as a percentage of the control (set to 100%).

### 2.7. Statistical Analysis

The experimental data are expressed as the means ± standard deviation (SD). Differences in the percentages among the experimental groups were compared via unpaired Student’s *t*-test using GraphPad Prism 6 [39]. The effect was considered significant when the *p*-value (*p*) was ≤ 0.05.

## 3. Results

### 3.1. Characterization and Analytical Determinations

**Figure 1:**
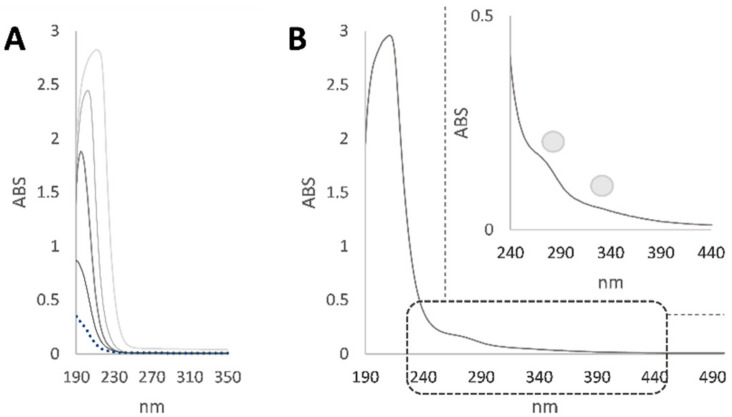
(**A**) UV spectra of lincomycin at different concentrations in the 25–500 mg/L range; (**B**) UV spectrum of tank water with an enlargement of the region between 240 and 440 nm.

Zebrafish were exposed to TiO_2_ NPs and lincomycin, and the effects in terms of genetic material alteration were assessed. Indeed, lincomycin and TiO_2_ NPs in the exposed organisms were indirectly assessed by estimating the lincomycin and nanoparticle content in the tank water. For this purpose, at the end of the exposure time, the tank water first underwent UV spectrophotometric analysis (Figure 1). 

The UV spectrum of the tank water showed a band at 203 nm and a shoulder at 277 nm. The first band was in accordance with lincomycin absorption, whereas the second one was attributable to TiO_2_ NPs (Figure 1). In fact, TiO_2_ NPs exhibited strong absorption in the UV region from 250 to 350 nm. In order to finely quantize the antibiotic and TiO_2_ NPs content in the water, HPLC DAD analysis was carried out; based on the acquired lincomycin calibration curve (y = 5.2582x + 42.55; R^2^ = 0.9971), it was found that the water contained 98.8 mg/L of lincosamides and 8.2 μg/L of TiO_2_ NPs. The pH measurement was in line with the preservation of the nanoparticles size. In fact, pH values ranged from 7.0 to 7.4 in all the experimental treatments. SEM analysis on dried test solutions previously performed showed the particle size was close to the manufacturers’ information (mean ± SEM, n = 80 images; 23.8 ± 73.5 nm) [22].

### 3.2. Cell Viability 

**Figure 2:**
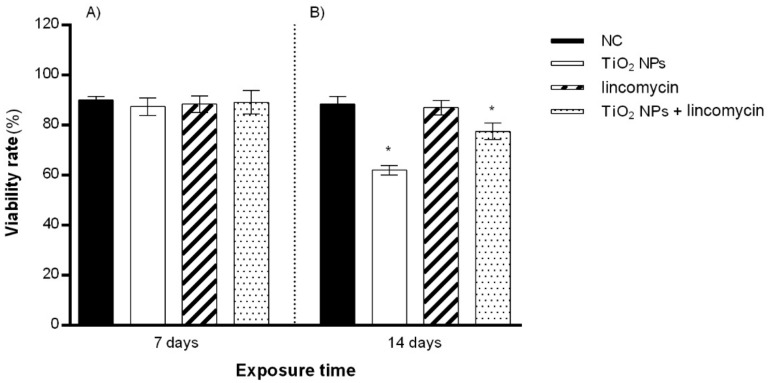
Percentage of live zebrafish blood cells (ordinate) after 7 days of exposure (**A**) and after 14 days of exposure (**B**) to TiO_2_ NPs alone, lincomycin alone, and TiO_2_ NPs and lincomycin in combination (abscissa). The dark bars are the negative control (NC); the white bars are 10 μg/L TiO_2_ NPs-treated specimens (TiO_2_ NPs); the striped bars are 100 mg/L lincomycin treated specimens (lincomycin); and the dotted bars are 10 μg/L TiO_2_ NPs + 100 mg/L lincomycin treated specimens (TiO_2_ NPs + lincomycin); * *p* ≤ 0.05 in comparison with the NC.

TiO_2_ NPs alone and in combination with lincomycin provoked a slight increase in zebrafish blood cell mortality after 14 days of exposure. Exposure to lincomycin alone did not induce a decrease in cell viability after 7 or 14 days of treatment (Figure 2).

### 3.3. DNA Fragmentation 

**Figure 3:**
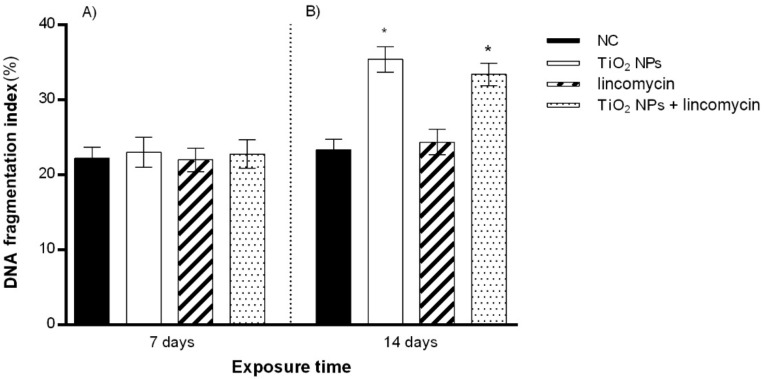
Percentage of DNA fragmentation in zebrafish blood cells (ordinate) after 7 days of exposure (**A**) and after 14 days of exposure (**B**) to TiO_2_ NPs alone, lincomycin alone, and TiO_2_ NPs and lincomycin in combination (abscissa). The dark bars are the negative control (NC); the white bars are 10 μg/L TiO_2_ NPs treated specimens (TiO_2_ NPs); the striped bars are 100 mg/L lincomycin treated specimens (lincomycin); and the dotted bars are 10 μg/L TiO_2_ NPs + 100 mg/L lincomycin treated specimens (TiO_2_ NPs + lincomycin); * *p* ≤ 0.05 in comparison with the NC.

**Figure 4:**
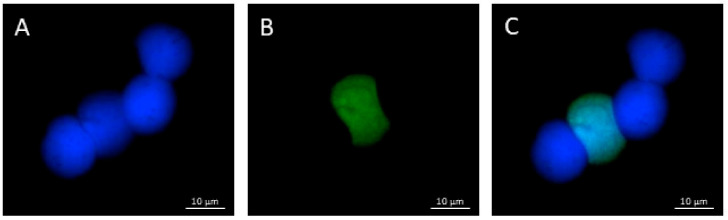
DNA fragmentation in zebrafish blood cells co-exposed to TiO_2_ NPs and lincomycin analyzed under a fluorescence microscope (Nikon Eclipse E-600) with different filters. (**A**) DAPI filter, which allows us to observe the nuclei (blue fluorescence); (**B**) fluorescein filter, which allows us to observe the nuclei with fragmented DNA (green fluorescence); (**C**) merged fluorescence image: the DAPI filter and the fluorescein filter combined.

After 14 days of TiO_2_ NP exposure, a statistically significant increase in the DNA fragmentation percentage was detected. Similarly, co-exposure to TiO_2_ NPs and lincomycin showed a statistically significant increase in the percentage of DNA fragmentation for the maximum exposure time tested (14 days). Lincomycin exposure did not provoke a statistically significant change in the percentage of DNA fragmentation for any of the exposure times (Figure 3 and Figure 4). 

### 3.4. RAPD PCR Fingerprints 

The amplification products obtained using RAPD PCR highlighted many bands of molecular size between 200 and 1500 base pairs (bp). 

After 7 days of exposure to TiO_2_ NPs alone, the RAPD PCR results showed the appearance of one new band at 650 bp and the simultaneous disappearance of three control bands at 200, 300, and 750 bp. After 14 days of TiO_2_ NPs treatment, seven polymorphic bands were found in the amplification patterns with respect to controls. 

Seven days of exposure to lincomycin induced only one band change compared to the control, while the appearance of two bands and disappearance of two bands were detected after 14 days of exposure. 

The samples co-exposed to the two molecules showed one polymorphic band after 7 days of exposure, whereas the samples treated for 14 days with TiO_2_ NPs and lincomycin exhibited the appearance of two additional bands and the disappearance of four control bands (Table 1).

### 3.5. Genomic Template Stability (GTS%)

The appearance/disappearance of the bands showed how exposure to TiO_2_ NPs alone induced a reduction in genomic stability starting from 7 days, which became very marked after 14 days.

**Figure 5:**
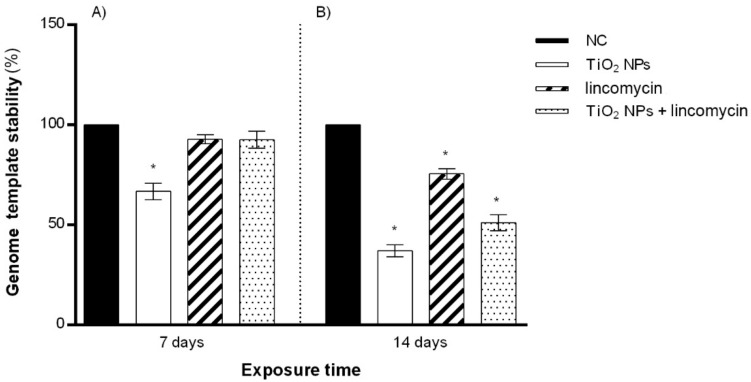
Percentage of genomic template stability (GTS%) in the zebrafish genome (ordinate) after 7 days of exposure (**A**) and after 14 days of exposure (**B**) to TiO_2_ NPs alone, lincomycin alone, and TiO_2_ NPs and lincomycin in combination (abscissa). The dark bars are the negative control (NC); the white bars are 10 μg/L TiO_2_ NPs treated specimens (TiO_2_ NPs); the striped bars are 100 mg/L lincomycin treated specimens (lincomycin); and the dotted bars are 10 μg/L TiO_2_ NPs + 100 mg/L lincomycin treated specimens (TiO_2_ NPs + lincomycin); * *p* ≤ 0.05 in comparison with the NC.

Lincomycin exposure reduced DNA stability only after 14 days, and the cotreatment with TiO_2_ NPs and lincomycin showed a reduction in GTS% for the maximum exposure time tested (Figure 5).

## 4. Discussion

Pollution is one of the foremost threats to health. According to the United Nations (UN), environmental pollution is responsible for at least 6 million deaths a year, and the presence of antibiotics dispersed in the environment is an important concern. The majority of the antibiotics are natural compounds that have been in contact with the environmental microbiota for millions of years and are biodegradable [40]. 

Although the antibiotic compounds are degraded in natural ecosystems, they are not excluded from classification as pollutants. Indeed, degradation is a slow process during the winter season due to low temperatures [41] and is also influenced by the composition and state of the soil, including moisture [42]. Moreover, all the drugs taken for therapeutic purposes and used for breeding purposes return to the environment through sewers and alluvial sediments [11,43]. The pharmacological molecules present in the wastewater can negatively interact with the biota DNA, causing damage to genetic heritage such as point mutations (insertions and deletions) and breaking of the double-stranded DNA, thus also affecting the subsequent offspring [31,44]. The persistence of antibiotics in the environment also makes them readily available for interaction with other contaminants that are ubiquitously present in it. Among such contaminants, NPs have been the object of numerous studies regarding their dangerous effects. Most of all, the risks are related to how difficult it is to analyze the behaviour of NPs and global applications. Exposure to NPs has grown considerably in the last century due to industrial development and the introduction of engineered NMs. NPs, due to their small size, can access cells’ mitochondria, the pulmonary alveoli, the cardiovascular system, and the central nervous system, determining pathologies associated with these [45,46,47]. In addition, strong toxic properties have been found at both the cellular and genomic levels through the determination of oxidative stress that induces inflammation, DNA damage, and mutations [48]. In particular, TiO_2_ NPs show toxicity related to their ability to form ROS after exposure to UV rays [49], to induce the breakage of DNA filaments and chromosomal alterations in several models [26,50,51,52]. NPs transport chemotherapeutic drugs and immune stimulators, but also substances that can be potentially toxic to human health, in a hidden way, without being identified as intruders by the human immune system. They are the only materials with this ability for now, and are thus referred to as a “Trojan horse”. With this property, these NPs are good carriers of substances of any kind towards the target cells, thus determining greater efficacy of the molecule since it does not spread throughout the body but acts directly on the intended target [53]. At the same time, transporting a potentially reactive substance to genetic material could lead to an increase in genotoxicity. In the literature, it is possible to find positive applications of these NPs, such as their ability to degrade antibiotics present improperly in environmental waters. This was evaluated in the work of Wypij et al., highlighting the antimicrobial and cytotoxic potential—markedly present when combined with antibiotics and antifungal agents—of silver nanoparticles (Ag + NPs) synthesised by *S. calidiresistens*, particularly from the IF11 and IF17 strains [54]. 

Our study evaluated the genotoxic potential of TiO_2_ NPs, lincomycin, and the effects of their association using *D. rerio* as an experimental model, a key component of the aquatic ecosystem chain. The evaluation of the effects caused by lincomycin and TiO_2_ NPs was carried out by estimating the molecular alterations in terms of DNA stability (GTS%), cell viability, and DNA strand breaks. TiO_2_ NPs showed genotoxic power against erythrocytes of zebrafish and induced mutations in the zebrafish genome, in accordance with our previous study [22]. This could be due to their nanometric dimensions and physicochemical characteristics that allow them to penetrate biological membranes, allowing direct damage to the DNA. In fact, NPs are able to cross cell membranes and be absorbed by a wide variety of types of mammalian cells, inducing cytotoxic and genotoxic damage [55]. 

However, from this study, particularly relevant were the results obtained following co-exposure to the two substances tested in vivo for 7 and 14 days at concentrations of 100 mg/L for lincomycin and 10 μg/L for TiO_2_ NPs. Although exposure to lincomycin alone only reduced the zebrafish genome stability and only at the maximum exposure time tested, its association with TiO_2_ NPs resulted in a decrease in cell viability and deleterious effects for the zebrafish genome in terms of DNA fragmentation, as well as a drastic GTS% reduction in comparison to lincomycin exposure alone. In particular, DNA damage was found after 14 days of co-exposure to the two substances, so a genotoxic effect can be hypothesised in the case of simultaneous intake for longer exposure times. 

Therefore, from the results obtained, it is possible to point out a synergistic interaction of TiO_2_ NPs and lincomycin. We obtained similar results in a human in vitro model cotreated with TiO_2_ NPs and lincomycin: their co-exposure determined the induction of the apoptotic process with DNA fragmentation in cultured human amniotic cells [32]. Our data represent an evolution of previous data that were limited to in vitro systems, allowing us to obtain a more complete profile regarding the effects of simultaneous exposure to antibiotics and NPs in an aquatic environment.

The results of this work confirm that the combination of TiO_2_ NPs and lincomycin is genotoxic to exposed aquatic organisms. On the other hand, lincomycin alone seems to be harmless for exposed fish; in fact, the polymorphic events found over prolonged times could be due to temporary and repairable damage [27], as cell viability and DNA breaks were never present in the genome of the same specimens exposed to the drug. 

From the UV spectra of lincomycin and TiO_2_ NPs, it emerged that the two substances dissolved in the fish breeding water interact, resulting in a synergistic effect, also potentially mediated by the establishment of weak intermolecular interactions influencing the behavior of NPs and, therefore, their activity. In fact, the genotoxicity values obtained following cotreatment with the two substances were lower than those resulting after individual TiO_2_ NPs treatment. The increased damage found with combined treatments compared to that with single lincomycin exposure allows us to hypothesise that NPs incorporate the drug and act as a vehicle for it into the target cells, probably increasing its bioavailability. However, it cannot be excluded that the interaction between TiO_2_ NPs and lincomycin at the tested concentrations causes the formation of a compound that reduces the genotoxicity of NPs rather than increases the genotoxicity of lincomycin. In fact, the methodology used to assess the amount of lincomycin removal and the amount remaining in the water is definitely not sufficiently accurate to reflect the amount available to the fish or the amounts of lincomycin in different organs of the fish. Therefore, further bioavailability and bioaccumulation studies are necessary to demonstrate whether NPs act as a vehicle for lincomycin into fish target organs and cells. 

However, the hypothesis that lincomycin had a stronger effect in the presence of TiO_2_ NPs due to more efficient transport of lincomycin to cells is supported by literature data where geno-/cytotoxic effects of other types of NPs in combination with antibiotics/antifungals were highlighted. Anyhow, it must be taken into account that the effect depends on the substance concentration, type of interaction, and exposure medium.

Recent scientific evidence has indicated that silver nanoparticles (AgNPs) can potentiate the effect of some antibiotics: the synergistic activity of AgNPs with conventional antibiotics against multiresistant Gram-positive and Gram-negative bacteria was evaluated, and it was shown that AgNPs, in combination with antibiotics, exhibit enhanced cytotoxic effects in eukaryotic cells [54,56].

Although the conjugation of NPs with active antimicrobial peptides and their ability to enhance antimicrobial effects can provide great advantages in the field of drug delivery and therapeutic applications [57] genotoxicity tends to increase following co-exposure, and this raises special concerns regarding the health of humans and animals exposed to both substances.

The presence of NMs, in addition to excessive antibiotics in the environment, allows these different molecules to interact to each other, causing harmful and synergistic effects on organisms. The major risk of combining these two contaminants is that, in addition to being ecotoxic, they can cause harm to human health through the food chain. Additionally, some antibiotics, such as lincomycin, are supplied in capsules constituted, in addition to gelatine, by TiO_2_ NPs: humans are thus directly exposed as a result of the assumption of this molecular complex.

In the light of this evidence, it is clear how dangerous direct and indirect exposure to these substances can be. 

However, the binding of the drug to the nanoparticles and their internalization to specific cells should be determined by the study of cellular biodistribution, subcellular localization of nanocarrier and intracellular uptake so, considering that confocal microscopy helps to ascertain the nanocarriers localization inside the cells additional, confocal microscopy analyses will be necessary [58]. Furthermore, transmission electron micrographs (TEM) will be useful to clarify whether cellular internalisation of the TiO_2_ NPs–lincomycin complex occurs and how this event could affect the expression of those genes involved in detoxification, apoptosis, or DNA repair, or TiO_2_ NPs act by release of Ti4+ ions, also using longer co-exposure times and different concentrations. Furthermore, whereas nanomaterials are known to induce developmental and reproductive toxicity [23], and considering that factors related to sex can affect the profile of biological responses, we believe that zebrafish life cycle assessment by studying vitellogenin, gonadal glands, sperm plasma membrane integrity and sex hormone production, taking into account difference in sex between the specimens studied, may represent a future perspectives for a more complete view of the effects of TiO_2_ NPs and lincomycin on exposed organisms.

## 5. Conclusions

Our data showed that simultaneous exposure to lincomycin and TiO_2_ NPs in an aquatic environment is harmful to the biota. Although the individual lincomycin did not induce irreparable damage to the DNA of the zebrafish, when interacting with TiO_2_ NPs it caused a genotoxic action. Considering the large spread of antibiotics and nanomaterials in wastewater, the risk that both pollutants can be present at the same time and interact with each other is very high; the resulting molecular complex could damage fish fauna and reach humans through the food chain with possible harmful consequences for health. Hence, if further bioaccumulation studies confirm the hypothesis of the function of TiO_2_ NPs carrying lincomycin into fish target organs and cells, monitoring the presence of both contaminants in aquatic environments will become necessary.

## Figures and Tables

**Table 1:** Molecular sizes (bp) of bands that appeared or disappeared after amplification with primer 6 in zebrafish blood cells DNA exposed to TiO_2_ NPs, lincomycin, or TiO_2_ NPs–lincomycin in combination. * Control bands are at 200, 240, 300, 320, 500, 550, 700, 720, 750, 800, 1000, and 1500 bp.

Substance Concentration	Exposure Days	Gained Bands (bp)	Lost Bands * (bp)
TiO_2_ NPs, 10 µg/L (*n = 20*)	7	650	200, 300, 750
	14	650, 850, 900	200, 300, 720, 750
Lincomycin, 100 mg/L (*n = 20*)	7	–	200
	14	350, 400	300, 320
TiO_2_ NPs, 10 µg/L + lincomycin, 100 mg/L (*n = 20*)	7	350	–
	14	350, 400	200, 320, 720, 750

## Data Availability

All the data generated or analyzed during this study are included.
